# Dent's disease

**DOI:** 10.1186/1750-1172-5-28

**Published:** 2010-10-14

**Authors:** Olivier Devuyst, Rajesh V Thakker

**Affiliations:** 1 Division of Nephrology, Université catholique de Louvain Medical School, Brussels, Belgium; 2 Academic Endocrine Unit, Nuffield Department of Medicine, Oxford Centre for Diabetes, Endocrinology and Metabolism (OCDEM), University of Oxford, Oxford, UK

## Abstract

Dent's disease is a renal tubular disorder characterized by manifestations of proximal tubule dysfunction, including low-molecular-weight proteinuria, hypercalciuria, nephrolithiasis, nephrocalcinosis, and progressive renal failure. These features are generally found in males only, and may be present in early childhood, whereas female carriers may show a milder phenotype. Prevalence is unknown; the disorder has been reported in around 250 families to date. Complications such as rickets or osteomalacia may occur. The disease is caused by mutations in either the *CLCN5 *(Dent disease 1) or *OCRL1 *(Dent disease 2) genes that are located on chromosome Xp11.22 and Xq25, respectively. *CLCN5 *encodes the electrogenic Cl^-^/H^+ ^exchanger ClC-5, which belongs to the CLC family of Cl^- ^channels/transporters. *OCRL1 *encodes a phosphatidylinositol bisphosphate (PIP_2_) 5-phosphatase and mutations are also associated with Lowe Syndrome. The phenotype of Dent's disease is explained by the predominant expression of ClC-5 in the proximal tubule segments of the kidney. No genotype-phenotype correlation has been described thus far, and there is considerable intra-familial variability in disease severity. A few patients with Dent's disease do not harbour mutations in *CLCN5 *and *OCRL1*, pointing to the involvement of other genes. Diagnosis is based on the presence of all three of the following criteria: low-molecular-weight proteinuria, hypercalciuria and at least one of the following: nephrocalcinosis, kidney stones, hematuria, hypophosphatemia or renal insufficiency. Molecular genetic testing confirms the diagnosis. The differential diagnosis includes other causes of generalized dysfunction of the proximal tubules (renal Fanconi syndrome), hereditary, acquired, or caused by exogenous substances. Antenatal diagnosis and pre-implantation genetic testing is not advised. The care of patients with Dent's disease is supportive, focusing on the treatment of hypercalciuria and the prevention of nephrolithiasis. The vital prognosis is good in the majority of patients. Progression to end-stage renal failure occurs between the 3^rd ^and 5^th ^decades of life in 30-80% of affected males.

## Disease name and synonyms

Dent disease 1 (OMIM #300009)

X-linked recessive nephrolithiasis (OMIM #310468)

X-linked recessive hypercalciuric hypophosphataemic rickets (OMIM #300554)

Low-molecular-weight proteinuria with hypercalciuria and nephrocalcinosis (OMIM #308990)

Dent disease 2 (OMIM #300555)

## Definition and epidemiology

Dent's disease (OMIM #300009) refers to a heterogeneous group of X-linked disorders that have previously been reported as X-linked recessive nephrolithiasis, X-linked hypercalciuric hypophosphataemic rickets, or idiopathic low-molecular-weight proteinuria with hypercalciuria and nephrocalcinosis [1-6]. The disease is characterized by manifestations of proximal tubule (PT) dysfunction associated with hypercalciuria, nephrolithiasis, nephrocalcinosis, and progressive renal failure [7]. Low-molecular-weight (LMW) proteinuria represents the most consistent manifestation of Dent's disease, detected in almost all affected males and obligate female carriers. There is considerable inter- and intra-familial variability in the other manifestations of PT dysfunction, which may cause a renal Fanconi syndrome with hypophosphataemic rickets, as well as in the extent of nephrocalcinosis/nephrolithiasis. Dent's disease is a rare disorder, with around 250 affected families reported to date [8,9].

## Clinical description

Dent's disease is characterized by PT dysfunction and LMW proteinuria, associated with hypercalciuria, nephrolithiasis, nephrocalcinosis, and progressive renal failure. Dent's disease may also be associated with aminoaciduria, phosphaturia, glycosuria, uricosuria, kaliuresis, and impaired urinary acidification, and is often complicated by rickets or osteomalacia [4]. These features are generally found in males only, who may have manifestations of the disease from early childhood [7,8,10]. These patients may present with bone pain and difficulty in walking due to rickets, or symptoms of renal stones such as abdominal pain and haematuria. Occasionally, patients are referred as a result of the fortuitous discovery of biological manifestations of PT dysfunction, including LMW proteinuria. LMW proteinuria, which is characterised by the excretion of proteins such as α1 and β2 microglobulins, retinol-binding protein (RBP), Clara cell protein, and vitamin D binding protein, is found in approximately 99% of affected males. It has been hypothesized recently that the urinary loss of RBP may cause episodic night blindness in some patients [11]. Hypercalciuria and nephrocalcinosis are also highly prevalent and occur in 95% and 75% of affected males respectively, although there is considerable inter- and intra-familial variability in the occurrence of nephrolithiasis which occurs in approximately 50% of affected males. Progression to end-stage renal failure occurs between the 3^rd ^and the 5^th ^decades of life in 30-80% of affected males [7]. These manifestations of Dent's disease may occur occasionally in females. For example, the milder features of LMW proteinuria and hypercalciuria are found in approximately 70% and 50% of females carriers, respectively, whilst the more severe manifestations of nephrolithiasis have been reported in only 10 females and end-stage renal failure has been reported in only 1 female [8]. Like other tubulopathies, Dent's disease has been associated with rare cases of proteinuria and biopsy-proven focal glomerulosclerosis [12]. The occurrence of these predominantly renal manifestations and their association with *causative *mutations in ClC-5 (see below) is referred to as Dent disease 1.

Some patients with Dent's disease have been observed to have extra-renal manifestations such as mild intellectual impairment [1], hypotonia and cataract, and such patients have been reported to share mutations in OCRL1 with the oculo-cerebrorenal syndrome of Lowe [9,13]. The occurrence of these extra-renal manifestations with mutations relating to Lowe syndrome is referred to as Dent disease 2 [13]. To date, around 20 patients with Dent disease 2 have been reported, all of whom have hypercalciuria and LMW proteinuria. In addition, these patients may also have nephrocalcinosis, nephrolithiasis, haematuria, hypophosphataemia and/or renal insufficiency. Only a minority (approximately one-fourth) of these patients have been observed to have mild intellectual deficit, hypotonia and sub-clinical cataract. It is important to note that the presence of intellectual impairment and sub-clinical cataract were so mild as to dissuade the clinicians from considering a diagnosis of Lowe's syndrome, which is characterised by congenital cataracts, delayed motor milestones, some degree of intellectual impairment in almost all affected males, growth retardation, rickets and renal proximal tubulopathy. Moreover, the patients with Dent disease 2 and mild intellectual deficit were adults, who had not, over time, developed more overt features of Lowe's syndrome [9,13].

## Genetics

Dent's disease may be caused by either inactivating mutations in *CLCN5 *(OMIM #300008), which is located on chromosome Xp11.22 and encodes a 746 amino-acid electrogenic Cl^-^/H^+ ^exchanger (ClC-5) [5,14], or the *OCRL1 *gene, which is located on chromosome Xq25 and encodes the phosphatidylinositol 4,5-biphosphate 5 -phosphatase OCRL1 [13]. ClC-5 contains 18 α-helices, with two phosphorylation and one N-glycosylation sites. Structural studies have revealed that the protein forms diamond-shaped homodimers composed of two repeated halves that span the membrane in opposite orientations. Each subunit has its own pore responsible for the selective coupling of the Cl^- ^flux to H^+ ^counter-transport [15]. The total number of reported *CLCN5 *mutations is 148, and these are scattered throughout the coding region, with no evidence for major mutational hot spots [8]. Furthermore, there appears to be no correlation between the mutations and phenotypes and/or between the presence or absence of a *CLCN5 *mutation and the Dent's disease phenotype. Of the total 148 *CLCN5 *mutations, approximately 36% are nonsense mutations, 33% are missense mutations, 14% are frameshift deletions, 5% are frameshift insertions, 3% are donor splice site mutations, 3% are acceptor splice site mutations, 2% are intragenic deletions, 1% are novel splice site mutations, 1% are complete deletions of the gene, 1% are in-frame insertions, and 1% are in-frame deletions. The majority are predicted to result in truncated or absent ClC-5 protein, which would lead to complete loss of antiporter function. Indeed heterologous expression of these Dent's disease *CLCN5 *mutants, in either *Xenopus laevis *oocytes or HEK293 cells, has revealed that the majority of *CLCN5 *mutations lead to a loss of Cl^- ^conductance [5]. Further detailed studies of the *CLCN5 *missense mutations have revealed that these may lead to one of three abnormalities; endoplasmic reticulum retention and degradation of ClC-5, defective endosomal acidification, or altered endosomal distribution of ClC-5 but not defective endosomal acidification [16]. Of note, the majority of the missense mutations are clustered at the interface between the two subunits, emphasizing the functional importance of ClC-5 homodimerisation [17]. Furthermore, genetic inactivation of the *Clcn5 *gene in mice mimics the severe PT dysfunction observed in Dent's disease, including hypercalciuria and nephrocalcinosis (see below).

Approximately 40% of patients with Dent's disease do not have *CLCN5 *mutations, even though they are clinically indistinguishable from those that have *CLCN5 *mutations [8]. Twenty of these patients have been reported to have *OCRL1 *mutations [9,13], although it is important to note that none of these had the severe cataracts or intellectual deficit that is typically found in patients with Lowe syndrome. Consistent with these phenotypic differences, it is interesting to note that the *OCRL1 *mutations associated with Dent disease 2 do not overlap with those causing Lowe syndrome. All of the *OCRL1 *missense mutations associated with Dent's disease occur in the 5' region of the gene (exons 4 to 15) and involve the phosphatidylinositol phosphate 5-phosphatase domain of the OCRL1 protein, whilst the truncating mutations are in the first seven exons or intron 7. By contrast, the *OCRL1 *mutations that are found in Lowe syndrome patients occur primarily in exons 9-22, which encode the 3 large functional domains [9]. A model in which a reduced but functioning form or isoform of OCRL1 protein is expressed in Dent disease 2, but not Lowe syndrome, has been proposed to explain the milder phenotypic features observed in the former patients [9]. Thus, there is genetic heterogeneity for Dent's disease, with approximately 50-60% of patients having *CLCN5 *mutations (Dent disease 1), ~15% harbouring *OCRL1 *mutations (Dent disease 2) and the remaining 25-35% of patients having neither *CLCN5 *nor *OCRL1 *mutations but possibly defects in other genes. The possibility that these other genes may encode some of the proteins (e.g. ClC-4 and cofilin) that interact with ClC-5 [18] has been investigated but no mutations in *CLCN4 *or *COFILIN *were identified [8].

## Pathophysiology

**Figure 1:**
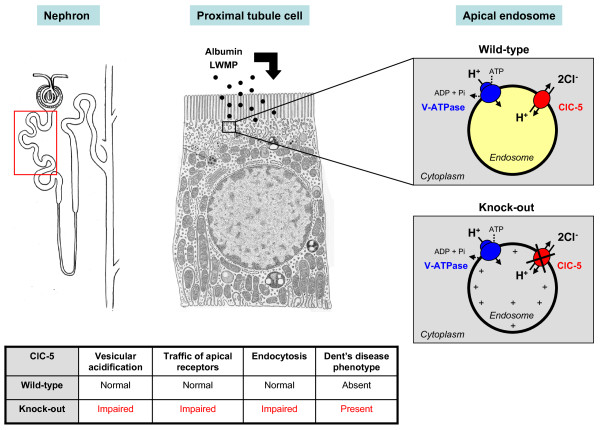
**Role of ClC-5 and pathophysiology of Dent's disease**. The epithelial cells lining the proximal tubule segments of the nephron (boxed area) are characterized by their capacity to reabsorb albumin and low-molecular-weight proteins (LMWP, black dots) that are ultrafiltered by the glomerulus. The intense endocytic activity in proximal tubule cells involves coated pits and coated vesicles, followed by early endosomes that form recycling endosomes or mature to late endosomes and lysosomes. Endosomal acidification (up to pH 5.0), that is necessary for dissociation of the ligand-receptor complex, recycling of receptors to the apical membrane, and progression of ligands into lysosomes, is achieved by ATP-driven transport of cytosolic H^+ ^through the V-ATPase. In the apical endosomes, wild-type ClC-5 Cl^-^/H^+ ^exchanger (*upper panel*) provides a countercurrent for the proton pump, which facilitates vesicular acidification. In ClC-5 knock-out endosomes (*lower panel*), the functional loss of ClC-5 impairs vesicular acidification by accumulating positive charges in the lumen. Defective endocytosis ensues, with a generalized dysfunction of proximal tubule cells and manifestations of renal Fanconi syndrome.

The complex phenotype of Dent disease 1 is probably explained by the predominant expression of ClC-5 in the PT segments, with more discrete expression in the thick ascending limb (TAL) of Henle's loop and the α-type intercalated cells (IC) of the collecting ducts of the kidney [19]. In PT cells, ClC-5 co-distributes with the vacuolar H^+^-ATPase (V-ATPase) in early endosomes [19,20], which are responsible for the reabsorption and processing of albumin and LMW proteins that are filtered by the glomerulus (Figure 1). These vesicles belong to the receptor-mediated endocytic pathway, which involves the multiligand receptors, megalin and cubilin, located at the apical brush border of PT cells [21]. Progression along the endocytic apparatus depends on endosomal acidification, driven by the V-ATPase and requiring a parallel Cl^- ^conductance to maintain electroneutrality. It has long been assumed that ClC-5 could provide such an electrical shunt to neutralize the H^+ ^gradient. Accordingly, the loss of the endosomal Cl^- ^conductance mediated by ClC-5 would impair vesicular acidification, causing dysfunction of PT cells. Two independent strains of ClC-5 knock-out (KO) mice have been generated, which both recapitulate the major features of Dent's disease including LMW proteinuria and other manifestations of PT dysfunction [22,23]. Furthermore, *in vitro *experiments have shown a decreased acidification of early endosomes in ClC-5-deficient mice [24,25].

However, ClC-5 is a 2Cl^-^/H^+ ^exchanger rather than a Cl^- ^channel [14], and the relevance of this exchange activity for Dent's disease was unknown. To address that important issue, Jentsch and colleagues engineered a knock-in (KI) mouse harbouring a point mutation in a critical glutamate residue which converts the exchanger into an uncoupled Cl^- ^channel that should facilitate endosomal acidification. They then compared these KI mice with the conventional ClC-5 KO mouse [26]. As expected, acidification of the renal endosomes from wild-type and KI mice was normal, but severely impaired in KO mice. However, despite normal endosomal acidification, KI mice showed the same renal phenotype than KO mice and patients with Dent's disease, including LMW proteinuria, hyperphosphaturia and hypercalciuria. Furthermore, both the KI and KO mouse showed impaired PT endocytosis, indicating that PT dysfunction in Dent's disease may occur despite normal acidification of the endosomes. These findings suggest a role for a reduced endosomal Cl^- ^accumulation in Dent's disease and, by extension, point to the importance of Cl^- ^concentration for organelle physiology [26].

Studies in mice have demonstrated that inactivation of ClC-5 is associated with a severe trafficking defect in PT cells, with loss of megalin and cubilin at the brush border, subsequent loss of their ligands in the urine, and impaired lysosomal processing [22,23,27]. Since the megalin/cubilin complex mediates the reabsorption of the vitamin D-binding protein, the 25(OH)-vitamin D3 and parathyroid hormone (PTH) that are ultrafiltrated by the glomerulus, the urinary loss of these mediators could potentially lead to opposite effects in PT cells, resulting in variable levels of active 1,25(OH)_2_-vitamin D_3 _levels in the serum [28]. Such variability could explain why renal hypercalciuria and kidney stones are present in one strain of ClC-5 KO mouse [23] but not in the other [22], potentially reflecting the phenotype variability observed in patients harbouring ClC-5 mutations [7]. Recently, Gailly et al. showed that the deletion of ClC-5 in mouse and human PT cells is associated with increased cell proliferation, oxidative stress and the specific induction of type III carbonic anhydrase [29]. Furthermore, ClC-5 inactivation is associated with impaired lysosome biogenesis, which also contributes to defective endocytosis and urinary loss of LMW ligands and lysosomal enzymes [30]. It must be emphasized that other inherited disorders targeting the PT cells, such as lysosomal storage disorders (cystinosis) or mitochondrial cytopathies, may result in PT dysfunction similar to that observed in Dent's disease [31].

The potential roles of ClC-5 in the TAL (involved in the urinary concentration mechanism and the regulated reabsorption of divalent cations) [32] and in the α-type IC (responsible for distal urinary acidification) remain to be defined [33]. Of interest, *CLCN5 *mutations have not been detected in patients with idiopathic hypercalciuria and in the hypercalciuric stone-forming (GHS) rat strain [34]. The hypercalciuria observed in patients with Dent's disease and some ClC-5-deficient mice may be secondary to the PT dysfunction (urinary loss of vitamin D binding protein and reduced phosphate absorption, leading to increased 1,25(OH)_2_-vitamin D_3 _synthesis) or, at least in part, caused by the functional loss of ClC-5 in the TAL. A small fraction of patients with Dent's disease may have nephrocalcinosis without hypercalciuria [10], which could indeed reflect the fact that ClC-5 is distributed in several nephron segments that can contribute to the genesis of kidney stones through different mechanisms. For instance, it has been suggested that collecting duct cells lacking ClC-5 may show an impaired ability of internalization of calcium crystals adhering to apical cell surface [35]. In summary, we can hypothesize that the functional loss of ClC-5 is essentially reflected by manifestations of PT dysfunction and may contribute to the genesis of kidney stones in different ways, reflecting its involvement in specific tubular functions. The issue is further complicated by the existence of a significant inter- and intra-familial variability in the manifestations of nephrocalcinosis and kidney stones.

Although ClC-5 mRNA and protein are detected in rodent intestine [36] and thyroid [37], no clear phenotype related to these tissues has been reported in patients. Of note, ClC-5 is highly expressed in the mouse thyroid, located in various endosomes at the apical pole of the thyrocytes. Mice lacking ClC-5 develop a euthyroid goiter, which results from impaired apical iodide efflux (secondary to down-regulated pendrin) rather than defective apical endocytosis [37].

The phenotype of Dent disease 2 due to *OCRL1 *mutations may in part be attributed to the role of *OCRL1 *in lysosomal trafficking and endosomal sorting. *OCRL1 *encodes a member of the type II family if inositol polyphosphate 5-phosphatases [38]. These enzymes hydrolyze the 5-phosphate of inositol 1, 4, 5-trisphosphate and of inositol 1,3,4,5-tetrakisphosphate, phosphatidylinositol 4,5-bisphosphate, and phosphatidylinositol 3,4,5-trisphosphate, thereby presumably inactivating them as second messengers in the phosphatidylinositol signalling pathway [39]. The preferred substrate of OCRL1 is phosphatidylinositol 4,5-bisphosphate (PIP_2_), and this lipid accumulates in the renal PT cells of patients with Lowe syndrome [39]. OCRL1 is localised to lysosomes in renal PT cells and to the trans-Golgi network in fibroblasts. This localisation is consistent with the role of OCRL1 in lysosomal enzyme trafficking from the trans-Golgi network to lysosomes, and the activities of several lysosomal hydrolases are found to be elevated in the plasma of affected patients [40]. OCRL1 has also been shown to interact with clathrin and indeed co-localises with clathrin on endosomal membranes that contain tranferrin and mannose 6-phosphate receptors [41]. Mannose 6-phosphate receptor-bound lysosomal enzymes are recruited by appendage (AP) subunits and Golgi-localised binding proteins into clathrin-coated vesicles that transport them from the trans-Golgi network to endosomes [41]. More recently, Erdmann et al. showed that OCRL1 plays a role in the early endocytic pathway, by interacting with the Rab5 effector APPL1 [42]. Thus, it seems likely that the *OCRL1 *mutations in Lowe syndrome patients result in OCRL1 protein deficiency, which leads to disruptions in the endosomal and/or lysosomal trafficking. This abnormality is similar to that observed in Dent disease 1, and it seems that Dent's disease therefore may be due to abnormalities in either endosomal acidification and sorting, or lysosomal trafficking. It must be noted that the targeted disruption of the murine ortholog for OCRL1 does not cause Lowe syndrome, because *Ocrl1 *deficiency is complemented in mice by inositol polyphosphate 5-phosphatase (*Inpp5b*) [43]. Thus, no mouse model recapitulating Lowe syndrome caused by the deficiency in OCRL1 is available.

## Diagnosis

The clinical diagnosis of Dent's disease is based on the presence of all three of the following criteria: (i) LMW proteinuria (elevation of urinary excretion of β2-microglobulin, Clara cell protein and/or RBP by at least 5-fold above the upper limit of normality); (ii) hypercalciuria (> 4 mg/kg in a 24 h-hour collection or > 0.25 mg Ca^2+ ^per mg creatinine on a spot sample); and (iii) at least one of the following: nephrocalcinosis, kidney stones, hematuria, hypophosphataemia, or renal insufficiency. The clinical diagnosis is supported by a history of X-linked inheritance of renal Fanconi syndrome and/or nephrolithiasis. The identification of mutation in either *CLCN5 *or *OCRL1 *confirms the diagnosis. However, some patients with *CLCN5 *mutations have been reported to have LMW proteinuria or hypercalciuria alone [34,44], and thus in the presence of an identified *CLCN5 *mutation, only one of the above clinical criteria may be sufficient to establish an affected status in an individual. It is important to note that the absence of clinical cataracts and the lack of severe intellectual deficit are key features that make a diagnosis of Dent disease 2, associated with *OCRL1 *mutations, more likely than a diagnosis of Lowe syndrome.

There have been few reports of renal biopsies in cases with proven *CLCN5 *mutations [45]. Light microscopy studies revealed progressive, non-specific lesions that include glomerular hyalinosis, tubular cell degeneration or atrophy, and mild interstitial fibrosis. Of interest, these kidneys invariably showed hyaline casts that were sometimes calcified, located in the outer medulla and presumably the first manifestations of nephrocalcinosis. By contrast, electron microscopy did not reveal any ultrastructural abnormalities in PT cells [45].

## Differential diagnosis

The differential diagnosis of Dent's disease includes the other causes of generalized PT dysfunction (renal Fanconi syndrome), that can be inherited, acquired or caused by exogenous substances [31,46] (Appendix 1).

## Genetic counselling

Both forms of Dent's disease are X-linked, and males, who are hemizygous, are affected more severely. Females, who are usually only mildly affected, are carriers and will transmit the disease to half of their sons whereas half of their daughters will be carriers. All the daughters of affected males will be carriers as they will have inherited the X chromosome harbouring the mutation, but all the sons of affected fathers who will have inherited the Y chromosome and not the X chromosome, will be normal. In approximately 10% of patients, Dent's disease occurs *de novo *and a family history is absent, but the disease will be transmitted as an X-linked trait to subsequent generations. If the mother or siblings of a patient with Dent's disease are eager to know their genetic status and risk for developing the disease, then mutational analysis of *CLCN5 *and/or *OCRL1*, using leukocyte DNA can be undertaken. However, it is important to note that it may not be routinely available in all genetic diagnostic laboratories. Although technically feasible, antenatal diagnosis and pre-implantation genetic testing for Dent's disease is not advised, and as yet has not been requested, because the vital prognosis in the majority of patients is good and there is no evidence for a genotype-phenotype correlation. Indeed the severity of the disease may vary considerably in individuals from the same family [10,34,47].

## Treatment

In the absence of therapy targeting the molecular defect, the current care of patients with Dent's disease is supportive, focusing on the prevention of nephrolithiasis. Thiazide diuretics can be used to treat hypercalciuria [48,49] although significant adverse events, including hypovolemia and hypokalemia related to the primary tubulopathy, have been reported [49]. Similarly, treatment of rickets with vitamin D must be cautious since it may increase hypercalciuria. Studies performed on ClC-5-deficient mice suggest that long-term control of hypercalciuria by a high citrate diet delays progression of renal disease even in the apparent absence of stone formation [50].

## Conclusions

Dent's disease is a renal tubular disorder caused by mutations in either the *CLCN5 *(Dent disease 1) or *OCRL1 *(Dent disease 2) genes that are located on chromosome Xp11.22 and Xq25, respectively. *CLCN5 *encodes the electrogenic Cl^-^/H^+ ^exchanger ClC-5, which is primarily located in the endosomes of the PT cells. The pathophysiology of the disease is essentially due to defective receptor-mediated endocytosis causing a generalized dysfunction of PT cells. *OCRL1 *encodes a PIP_2 _5-phosphatase and mutations are also associated with the oculo-cerebro-renal syndrome of Lowe, characterized by bilateral congenital cataract, severe mental retardation, and renal Fanconi syndrome. A few patients with Dent's disease do not harbour mutations in *CLCN5 *and *OCRL1*, pointing to the involvement of other genes. The care of patients with Dent's disease is supportive, focusing on the prevention of nephrolithiasis. The cautious use of thiazide diuretics has been suggested to treat the associated hypercalciuria.

## Abbreviations

IC: intercalated cell; LMW:low-molecular-weight; PIP_2_: phosphatidylinositol [4,5] bisphosphate; PT: proximal tubule; PTH: parathyroid hormone; RBP: retinol binding protein; TAL: thick ascending limb (of Henle's loop); V: ATPase; vacuolar H^+^-ATPase

## Competing interests

The authors declare that they have no competing interests.

## Authors' contributions

OD and RVT wrote the manuscript and approved its final version.

## Appendix 1. Differential diagnosis of Dent's disease: causes of renal Fanconi syndrome

### Inherited disorders

Dent disease

Lowe syndrome

Cystinosis

Galactosemia

Hereditary fructose intolerance

Glycogen storage disease (von Gierke disease)

Fanconi-Bickel syndrome

Tyrosinemia type I

Wilson disease

Mitochondrial diseases (cytochrome-c oxidase deficiency)

Idiopathic Fanconi syndrome

Sporadic Fanconi syndrome

### Acquired disorders

Glomerular proteinuria (nephrotic syndrome)

Light chain nephropathy (multiple myeloma)

Sjögren syndrome

Auto-immune interstitial nephritis

Acute tubulo-interestitial nephritis with uveitis (TINU)

Renal transplantation

Anorexia nervosa

### Exogenous substances

Drugs

Aminoglycosides, outdated tetracycline

Valproate, salicylate

Adefovir, cidofovir, tenofovir

Ifosfamide, cisplatin, imanitib mesylate

Chinese herbs (aristolochic acid)

Chemical compounds (paraquat, diachrome, 6-mercaptopurine, toluene, maleate)

Heavy metals (lead, cadmium, chromium, platinum, uramnium, mercury)
